# Taping Adjunct to Strengthening and Proprioception in a Hill-Sachs Lesion Patient: A Case Report

**DOI:** 10.7759/cureus.45816

**Published:** 2023-09-23

**Authors:** Sidra Ahmad Siraj, Pooja Dhage, Mitushi Deshmukh, Pratik R Jaiswal

**Affiliations:** 1 Musculoskeletal Physiotherapy, Ravi Nair Physiotherapy College, Datta Meghe Institute of Higher Education and Research, Wardha, IND

**Keywords:** hill-sachs lesion, scapular muscle rehabilitation., shoulder anterior dislocation, proprioception, taping

## Abstract

A Hill-Sachs lesion is a bony defect in the head of the humerus due to recurrent dislocation, which results in friction between the humeral head and the glenoid fossa. This recurrent incident of dislocation that occurs in the anterior direction eventually leads to a Bankart lesion (a defect in the glenoid rim). A 21-year-old male, a recreational football player, reported recurrent shoulder dislocation, complaining of pain and difficulty doing certain activities. He had hypermobility of the shoulder joint during joint play assessment. Proprioception is the sense of the position and movement of one's own body. Exercises that improve proprioception can help improve shoulder stability and reduce the risk of shoulder injuries. Proprioception has shown significant positive results in shoulder dislocations. A physiotherapy protocol was designed that included strengthening of shoulder and scapular musculatures, proprioceptive exercises, and plyometric exercises for developing agility. All these exercises were given with taping for the shoulder joint. Taping helps stabilize the shoulder and normalizes muscle function. With the help of physiotherapy, patients can avoid invasive procedures for restoring stability in non-traumatic recurrent shoulder dislocations.

## Introduction

Among all the shoulder dislocations, anterior dislocation has been shown to be the most common, i.e., 90%-95%, due to the anatomical structure and mobility of the shoulder joint. Shoulder dislocation is seen in 1%-2% of the population [1]. The prevalence of Hill-Sachs lesions during the first dislocation is 65% to 67%, and the percentage increases after recurrent dislocation to about 84% to 93% [2].

A Hill-Sachs lesion is a bony lesion of the posterior-superior-lateral head of the humerus due to recurrent shoulder instability or dislocation, mainly anterior dislocation or instability. The lesion is most evident on a radiograph of an internally rotated shoulder [3]. As the shoulder gets anteriorly dislocated, the capsular and labral structures are stretched and eventually torn up. As the head of the humerus further translates anteriorly, the humeral head comes into contact with the anterior portion of the glenoid cavity, causing a compression fracture of the humerus. The injured anterior soft tissue creates problems in recurrent shoulder instability patients as the static stabilizers become weakened. This makes the soft humeral head sustain injury due to repeated contact with the hard glenoid cavity [4]. The Hill-Sachs lesion commonly occurs with the Bankart lesion, which is an anterior capsule-labral [5].

The glenohumeral joint is an incongruent joint; for its stability, not only the bony structure but also other supports are required. This instability occurs due to the pull of gravity on the humerus. Thus, to maintain equilibrium, an equal and opposite force is needed. The rotator interval capsule is taut when the arm is held in a dependent position. The resultant vector formed from the rotator interval capsule and gravitational force creates a compressive force directed on the inferior border of the glenoid cavity, preventing inferior translation. In addition to this, the negative intracapsular pressure holds the humeral head against gravity, and the degree of upward inclination of the glenoid fossa produces a somewhat bony block against inferior translation. While heavy loading occurs, the supraspinatus activates and acts as a reinforcing force for the shoulder joint. In cases where dysfunction or paralysis occurs in this muscle, sustained loading on the shoulder results in gradual stretching of the rotator interval capsule, compromising joint stability [6].

The dynamic stabilization of the shoulder joint is provided by the deltoid and the rotator cuff muscles. The resultant force of the anterior and posterior deltoid is in line with the medial deltoid. Resolving the vector of the medial deltoid, the parallel vector should cancel out the gravitational force, thereby permitting the perpendicular force to act, causing rotation. This rotation causes impingement of the glenoid tubercle on the acromion. To avoid this, the gravitational force should not offset the parallel force because the force of the medial deltoid should be greater than gravity before any rotation can occur. Therefore, the deltoid alone cannot produce the desired force, so the rotator cuff muscle comes into play. The rotator cuff muscles blend into the glenohumeral capsule and reinforce it. The vector forces of teres minor, subscapularis, and infraspinatus have the same line of pull; this vector is resolved into a perpendicular force that tends to cause some rotation and also compresses the head of the humerus into the glenoid cavity. These muscles also play a crucial role in balancing out the superior translatory force of the deltoid muscle by producing an inferior translatory pull, which is generated by its parallel component [7].

During abduction, the infraspinatus and subscapularis muscles produce the abduction torque, while the teres minor muscles produce a later rotation, thus clearing the greater tubercle from beneath the acromion. Despite being a part of the rotator cuff muscles, the line of action of the supraspinatus muscle is in a superior direction and independently prevents the humeral head from slipping or translating inferiorly. It is capable of producing abduction independently in the first 15 degrees while simultaneously stabilizing the shoulder joint. The supraspinatus and rotator interval capsules are closely related to each other. Therefore, it contributes to passive stabilization. The resultant force vector of the supraspinatus, which produces a small superior translating force, is offset by the gravitational force, resulting in translatory equilibrium. The resultant force vector of the supraspinatus and gravitational force produces an inferiorly gliding force on the humerus during the abduction of the arm, preventing unwanted superior displacement [8].

Proprioception, or the sense of the position and movement of one's own body, is important for proper shoulder stability and function. Poor proprioception can lead to shoulder instability, as the shoulder joint may not be positioned properly or may move in an uncontrolled manner. This can also increase the risk of shoulder injuries and impair the performance of activities that require shoulder stability, such as lifting, throwing, and pushing [9].

Kinesio taping was originated by a Japanese chiropractor in 1980 [10]. Kinesio taping has been claimed to have four beneficial effects: decreasing pain, increasing vascular and lymphatic flow, normalizing muscle function, and correcting the position of the misaligned joint. Kinesio taping has been used as an adjuvant with physical therapy to improve rehabilitation. Taping for aligning joints improves stability and proprioception [11].

## Case presentation

A 21-year-old male patient, a recreational football player, complained of a left shoulder dislocation while playing football. The first time the patient experienced the anterior dislocation was in January 2022 while playing football. As narrated by the patient, he did not sustain any contact injuries; rather, the dislocation occurred while he was running. The pain at the time of dislocation on the numerical pain rating scale was 7/10 and quickly relieved, and the shoulder relocated on its own. The second dislocation happened after three months during the same event. Pain on the numeric pain rating scale (NPRS) was 6/10 and gradually decreased within two days, not hampering much of his activities. The third event took place in August, which was the most painful. With a rating of 8/10 on NPRS, he relocated his shoulder manually by himself. The pain persisted even after relocation and caused much difficulty in his activities. The pain lasted for about a week. Then he came to the physiotherapy department for consultation on October 20, 2022, when he had his third event of shoulder dislocation.

Assessment

Joint play is a movement that cannot be isolated independently by an individual. Joint play of the left shoulder showed hypermobility (Table 1).

**Table 1:** Joint play assessment

Joint play	Left shoulder
Anterior glide	Hypermobility
Caudal glide	Hypermobility

Special tests to check for instability and dislocation were evaluated (Table 2).

**Table 2:** Special test for shoulder instability and dislocation

Special test	Left shoulder joint
Anterior apprehension test	Positive
Andrews’ anterior instability test	Positive
Crank and relocation test	Positive
Load and shift test	Positive

The manual muscle resistance of the muscles of the shoulder had a strength of +4/5 on the Kendall grading scale.

Outcome measures

The shoulder pain and disability index (SPADI) questionnaire is used to measure the level of shoulder pain and disability percentage. It has been shown to be reliable for use in shoulder injuries (Table 3).

**Table 3:** Shoulder pain and disability index of the patient

Shoulder pain and disability index (SPADI)	Pre-physiotherapy	Post-physiotherapy week 3	Post-physiotherapy week 6
Pain scale	42%	24%	6.0%
Disability scale	28.75%	12.5%	2.5%
Total score	33.85%	16.9%	3.8%

Investigatory findings

**Figure 1:**
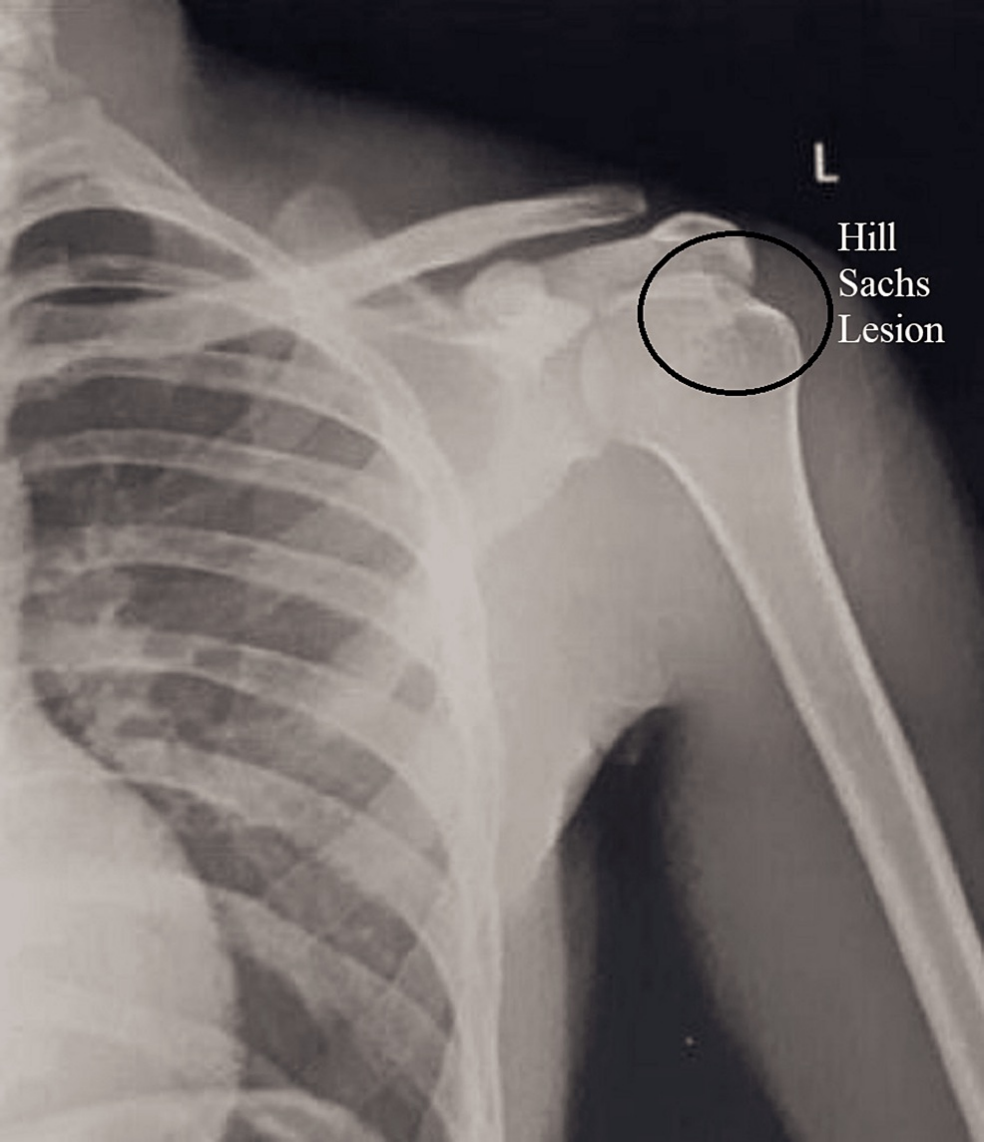
X-ray of the left shoulder

**Figure 2:**
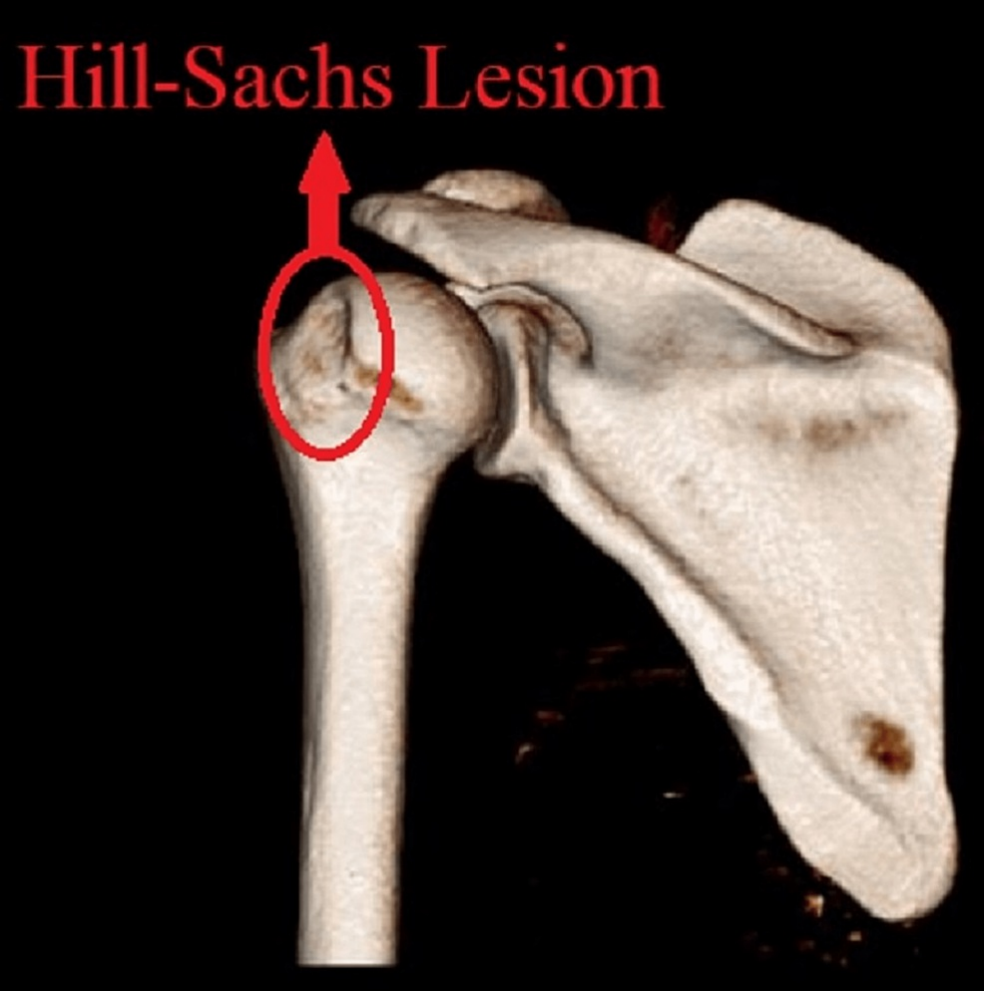
A 3D CT scan of the left shoulder joint

An X-ray (Figure 1) of the shoulder joint was done, but the lesion was not identifiable, so a 3D CT scan of the left shoulder was done, which showed a small lesion on the posterio-superior aspect of the head of the humerus (Figure 2).

Physiotherapy management

The patient was explained about the protocol regimen, and a week-wise protocol was designed for one and a half months (Table 4).

**Table 4:** Physiotherapy rehabilitation regimen of the patient

Week	Goals	Intervention	Regimen
1 - 2	Scapular muscle setting	Wall push-ups	10 repetitions, 2 sets, and a 1-minute break between each set with a 10-second hold.
		Belly press with a pillow between the abdomen and hand, and elbow out at the side.	
		Isometrics for shoulder extensors against a wall with elbow 90˚ flexed and neutral rotation	
		Isometrics of shoulder depressors with elbows at 90˚ against chair armrest	
		Isometrics for shoulder elevators	
		Isometrics for shoulder retraction and protraction	
		Isometrics for shoulder external rotation and internal rotation	
		Scapular squeeze	
	Shoulder and scapular muscle strengthening	Scapular upward rotation	15 repetitions and 2 sets, with a 1-minute break between each set with a half-kg weight cuff, and hold for 10 seconds. Shoulder range: 90˚.
		Prone lying shoulder abduction with lateral rotation in the I, Y, and T raises	
		Prone lying with arms at 90˚ abduction with internal rotation	
		Side-lying external rotation	
		Internal rotation with the shoulder abducted to 90˚	
		Sword pulls	
	Shoulder exercise in scaption	Shoulder abduction in prone lying in a pain-free range	
		Shoulder abduction in standing in a pain-free range	
2 - 4	Scapular muscle strengthening	Scapular upward rotation with resistance	25 repetitions and 3 sets, with one minute of rest between each set, using a 1.5 kg weight cuff and holding for 15 seconds. The shoulder range is above 90˚.
		Prone lying shoulder abduction with lateral rotation in I, Y, and T raises the upper, middle, and lower trapezius	
		Prone lying with arms at 90˚ abduction with internal rotation for rhomboids	
	Shoulder exercise in scaption	Shoulder abduction in prone lying in the pain-free range	
		Shoulder abduction in standing in the pain-free range	
	Shoulder range of motion exercises	Internal and external rotation in the scapular plane	25 repetitions, 3 sets, and a 1-minute break between each set with full shoulder range.
		Finger ladder	
		Sleeper stretch	
		Arm elevation with wand	
4 - 6	Plyometrics	Chair push-ups in the sitting position	Initial 20 repetitions and progress with repetition, speed, and resistance for 3 sets with a 1-minute break between each set.
		Horizontal ladder drill push-up	
		Falling push-up against a wall in standing at 90˚ abduction	
		Doorway fall push-up	
		Up and down ball rolls on the wall with both hands	
		Weight-bearing on the wobble board	
	Proprioceptive neuromuscular facilitation	D1 and D2 patterns with a theraband	
		D2 pattern with weight and whole body rotation from standing to a half-kneeling position	
		Push-ups with a therapy ball	
		Push-ups on the wobble board	

**Figure 3:**
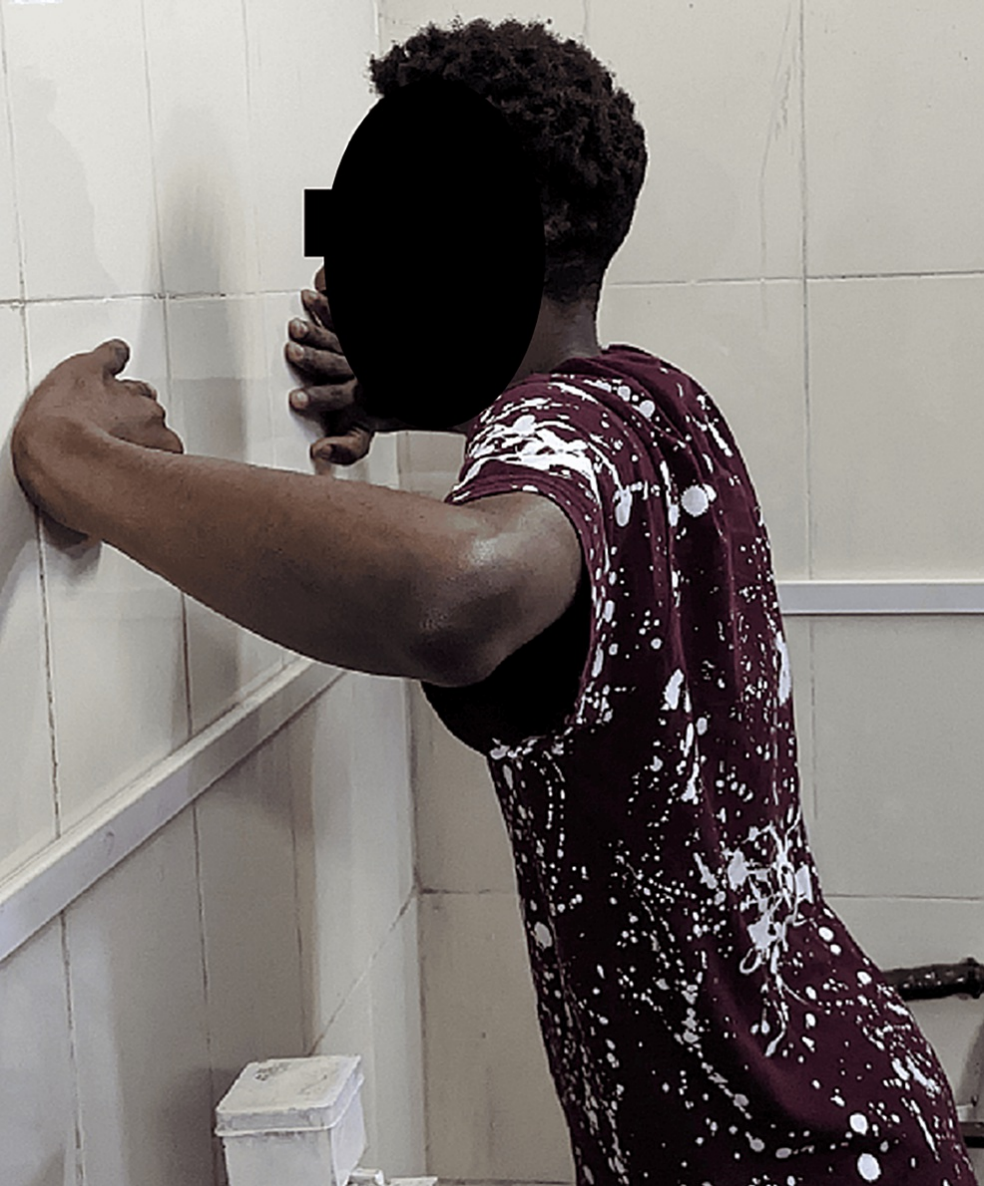
Wall push-up

**Figure 4:**
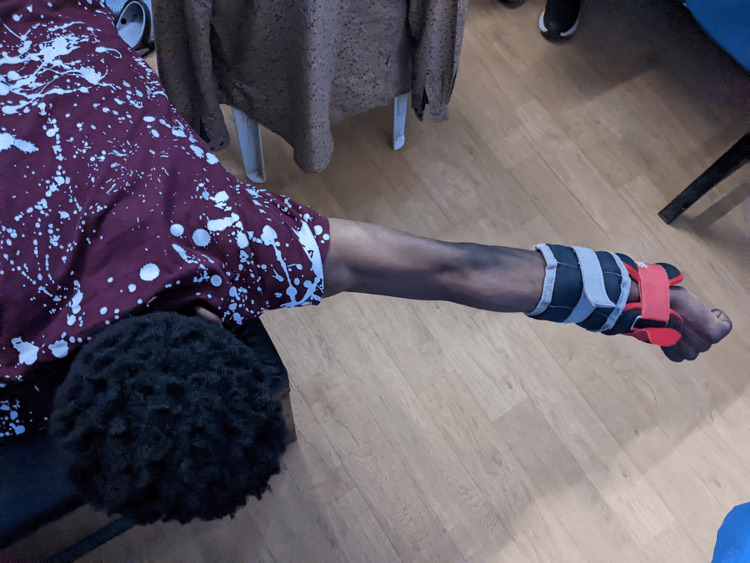
Scaption exercise in a prone lying position with internal rotation

**Figure 5:**
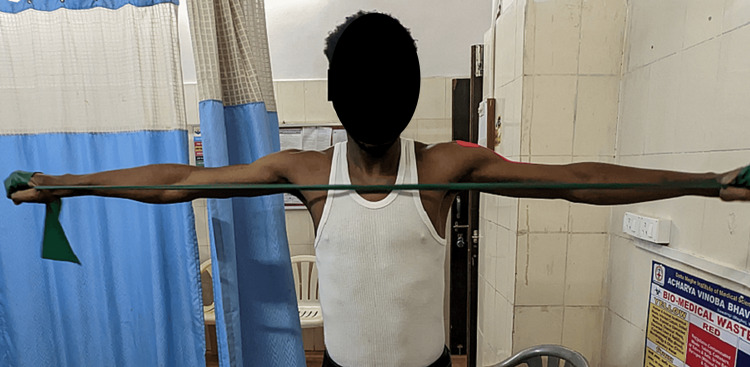
Abductor strengthening

**Figure 6:**
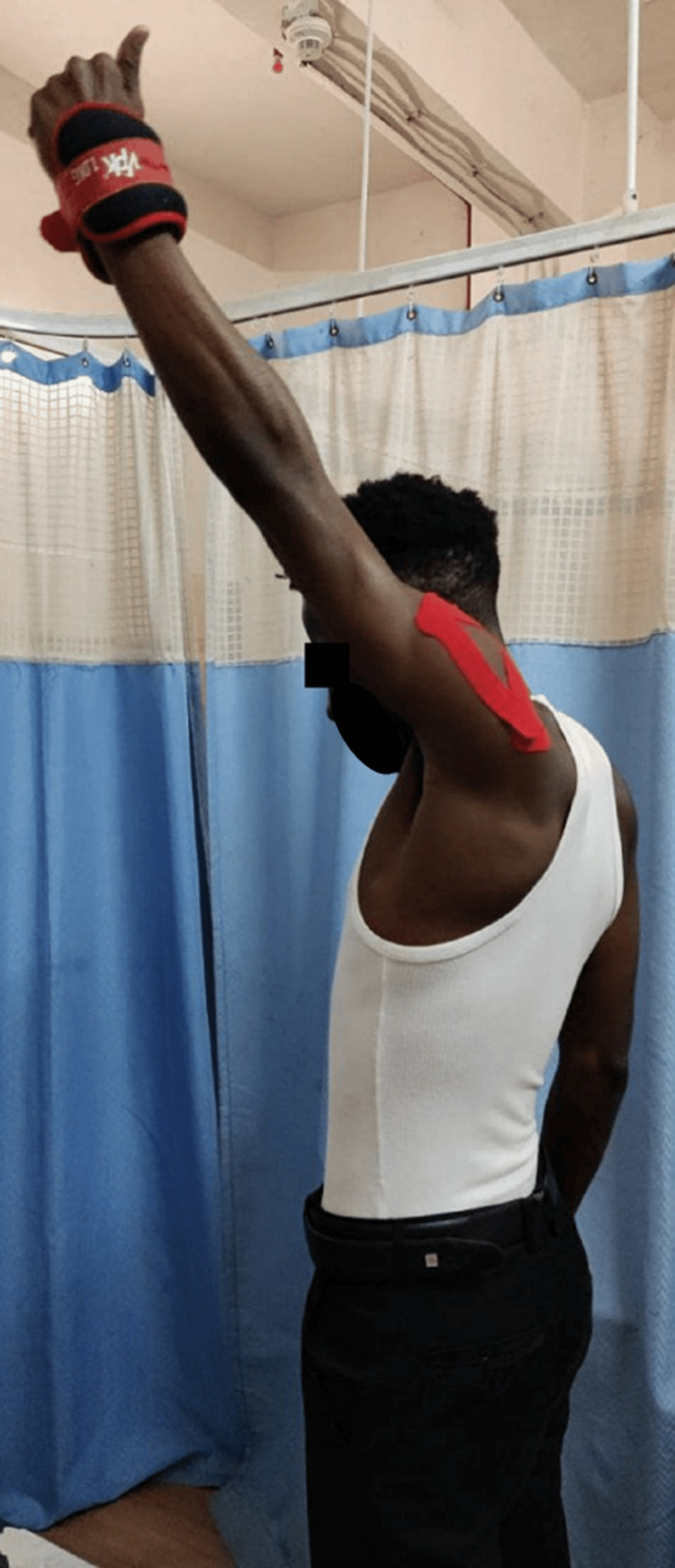
Abduction in the scaption plane

**Figure 7:**
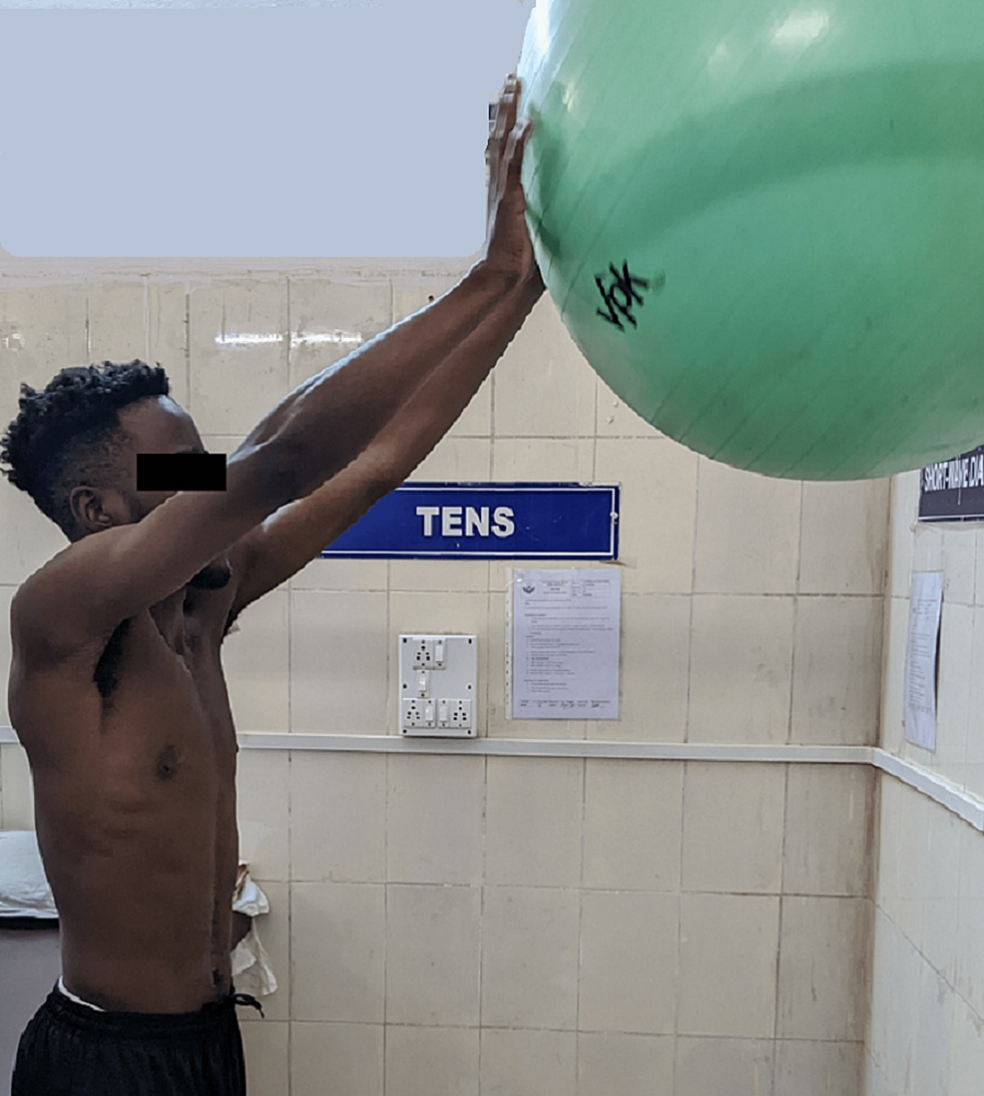
Wall ball rolls

Scapular setting exercises (Figure 3), strengthening of shoulder and scapular muscles (Figures 4, 5), exercise in scaption (Figure 6), and plyometric exercises (Figure 7) were prescribed.

Taping of the shoulder joint was done for the first four weeks during exercise for joint stability. The proprioceptive neuromuscular facilitation (PNF) principle has been utilized for increasing joint position sense, thereby providing joint stability.

## Discussion

Bateman et al., in their research on anterior shoulder instability due to atraumatic causes, said that strengthening exercises for the shoulder joint and scapular muscles and task-specific activities must be started immediately, keeping in mind that dislocation does not reoccur. Proprioception and high-speed polymetric exercises are all given for conservative management. Immobilization should be avoided, as it does not benefit instability recurrence but rather might lead to muscle atrophy [12]. According to Bateman et al., for atraumatic shoulder instability, Derby shoulder instability rehabilitation was followed, which focuses on proprioception, plyometrics, muscle strengthening, and muscle balance and works on deceleration of speedy movements. It showed significant benefits in function, stability, and pain [13]. McClure et al. studied the validity and reliability of different shoulder outcome measures, one of which was SPADI, and compared its original version, which was inconvenient to use, with the numerical version. The numerical version had 2.5 points more than the original one [14]. A study on scapular taping and its effect on shoulder joint repositioning during rehabilitation was conducted by Zanella et al.; they later concluded that clinicians should not worry about scapular taping interfering with shoulder elevation movements [15]. After six weeks of the physiotherapy protocol, the patient showed significant improvement, which was measured by the shoulder pain and disability index.

## Conclusions

This is a one-of-a-kind scenario because it includes physiotherapy rehabilitation that includes shoulder and scapular muscle strengthening and proprioceptive facilitation, combined with taping for the shoulder joint during exercise in the Hill-Sachs lesion. The patient showed significant improvement in both pain and activities. No dislocation episodes were observed during the one-month physiotherapy rehabilitation.
